# Focus on Chirality of HIV-1 Non-Nucleoside Reverse Transcriptase Inhibitors

**DOI:** 10.3390/molecules21020221

**Published:** 2016-02-16

**Authors:** Valeria Famiglini, Romano Silvestri

**Affiliations:** Institute Pasteur Italy—Fondazione Cenci Bolognetti, Dipartimento di Chimica e Tecnologie del Farmaco, Sapienza Università di Roma, Piazzale Aldo Moro 5, I-00185 Roma, Italy; valeria.famiglini@uniroma1.it

**Keywords:** chirality, HIV-1, reverse transcriptase, non-nucleoside inhibitor

## Abstract

Chiral HIV-1 non-nucleoside reverse transcriptase inhibitors (NNRTIs) are of great interest since one enantiomer is often more potent than the corresponding counterpart against the HIV-1 wild type (WT) and the HIV-1 drug resistant mutant strains. This review exemplifies the various studies made to investigate the effect of chirality on the antiretroviral activity of top HIV-1 NNRTI compounds, such as nevirapine (NVP), efavirenz (EFV), alkynyl- and alkenylquinazolinone DuPont compounds (DPC), diarylpyrimidine (DAPY), dihydroalkyloxybenzyloxopyrimidine (DABO), phenethylthiazolylthiourea (PETT), indolylarylsulfone (IAS), arylphosphoindole (API) and trifluoromethylated indole (TFMI) The chiral separation, the enantiosynthesis, along with the biological properties of these HIV-1 NNRTIs, are discussed.

## 1. Introduction

Human immunodeficiency virus type 1 (HIV-1) is the etiological agent of acquired immune deficiency syndrome (AIDS), a global pandemic disease which has claimed the lives of more than 34 million people so far. In 2014 alone, AIDS caused about 1.2 million deaths worldwide [1]. The treatment of HIV infected people is based on antiretroviral therapy (ART), also known as highly active antiretroviral therapy (HAART), which combines three or more drugs that suppress HIV replication [1]. ART can play an important role in preventing HIV transmission and post-exposure prophylaxis [2]. Thus far, there are no vaccines available for HIV. However, recent studies have shown that a vaccine can reduce the risk of HIV infection, and other novel vaccine strategies include broadly neutralizing antibodies that target a wide range of HIV strains [3,4]. Novel HIV vaccine candidates that are being developed, have shown to elicit strong protective antibody responses across the spectrum of HIV-1 strains. In the absence of such a vaccine, the nucleoside reverse transcriptase inhibitors (NRTI) can be an option for pre-exposure prophylaxis in preventing HIV infection in HIV-negative individuals [5].

The approved antiretroviral arsenal includes about thirty drugs falling in five main classes which control the levels of HIV-1 by targeting different steps of the viral life cycle. The drugs are nucleoside reverse transcriptase inhibitors that also include nucleotide agents, non-nucleoside reverse transcriptase inhibitors [6], viral maturation inhibitors (protease inhibitors), viral entry inhibitors including fusion inhibitors and co-receptor antagonists, and integration inhibitors [7]. These antiretroviral drugs may be administered singly or combined into multi-drug pharmaceutical formulations [8]. Targeting different steps of the viral life cycle has proven to be a successful strategy in the management of the HIV infection. The ART treatments suppress the viral replication and delay the progression of HIV infection, in particular in early stages of the disease. In most patients treated with ART therapy, the levels of plasma viremia remain below the limit of detection for at least six months [8]. However, some issues are still pending, namely the emergence of resistance even among drugs belonging to the same class (cross resistance), unwanted side effects and toxicity problems, which lead to failing to comply with the prescribed ART regimens [9,10,11,12,13,14,15]. Therefore there is a pressing need for new potent antiretroviral drugs with improved tolerability and better resistance profiles.

NNRTIs of HIV-1 are key drugs for the treatment of AIDS [16,17,18]. Thanks to their specificity for HIV-1, which makes them very selective inhibitors, NNRTIs became standard components of ART regimens [19,20]. NNRTIs show a wide variety of different structures. Despite the structural diversity, the NNRTIs show high specificity for the HIV-1 reverse transcriptase (RT), as they do not inhibit the HIV-2. The RT is a heterodimeric enzyme formed by the subunits p66 and p51 [21,22]. The NNRTIs bind to a common allosteric site of the RT, the non-nucleoside binding site (NNBS), that is a hydrophobic pocket located just 10 Å from the DNA polymerase active site, formed by residues Leu100, Lys101, Lys103, Val106, Thr107, Val108, Val179, Tyr181, Tyr188, Val189, Gly190, Phe227, Trp229, Leu234, Pro236 and Tyr318 in the p66 palm subdomain, and Glu138 of p51 [23]. The thumb is mainly in a closed position in unliganded RT. Upon NNRTI binding, the thumb moves to an upright position. The binding of the NNRTI to the RT may result in thumb mobility [18] and conformational changes of the catalytic site [24], hampering of the primer bond [25] and the thumb/finger connection [26], resulting in impairment of the enzyme catalysis. Some amino acids that make up the NNBS are part of the RT primer grip, the residues 227 and 235 in the palm domain position the primer end. Finally, the NNBS and the shift of the three-stranded β-sheet affect the position of residues D185 and D186 that coordinate the Mg^2+^ ions in the catalytic site [27]. Such changes may re-position the dNTP and impair chain elongation.

The ART regimens for a treatment-naive patient combines three or more antiretroviral drugs, generally two nucleoside reverse transcriptase inhibitors with a third non-nucleoside antiretroviral drug: a reverse transcriptase inhibitor, a protease inhibitor or an integrase strand transfer inhibitor [28]. NNRTI-based regimes increase age of patients living with HIV, have relatively few adverse [10] effects and better pharmacokinetic properties [29]. Major limitations of NNRTIs are the emergence of resistance and cross-resistance as a result of single and double amino acid mutations inside the NNBS of HIV-1 RT which hamper the binding of the NNRTI [30].

Chiral compounds often exhibit considerably different biological activities due to the highly specific nature of the chiral ligand-recognition site interaction [31,32]. The enantiomers, along with the corresponding racemates, require specific biological, pharmacological and toxicological studies before being launched in clinical practice [33]. Since 1992, the US Food and Drugs Administration (FDA) [34] and the European Committee for Proprietary Medicinal Products have required manufacturers to research and characterize each enantiomer in all drugs proposed to be marketed as a mixture. Therefore, racemic drugs ceased to be a rational commercial option, and academia and drug firms have been developing synthetic procedures and chiral separation techniques to achieve enantiomerically pure biologically active compounds [35].

The enantioselectivity plays an important role for the binding of antiviral agents to the asymmetric RT enzyme of HIV-1. The RT can incorporate enantiomeric nucleotides of both D and L series (while cell DNA polymerase cannot) as well as triphosphate analogues of l-nucleosides [36,37]. For example, the 3′-hydroxyl group of l-(β)-ribose is responsible for chiral discrimination between d- and l-enantiomers of deoxy- and dideoxy-nucleoside analogues [38]. The l-enantiomer of 2′,3′-dideoxycytidine (DDC) and its 5-fluoro congener (l-FDDC) showed to inhibit selectively HIV-1 *in vitro* [39]. β-l-5-fluorocytosine derivatives were found to be potent anti-HIV compounds [40]; (−)-FTC was also found to be a potent inhibitor of hepatitis B virus replication, while the d-isomer was found to be considerably less active against hepatitis B virus [41]. This paper provides an updated review that covers recent research on resistance to NNRTIs of approved and investigational drugs. We focused particularly on the chemical modification of chiral atoms that to our knowledge was not exhaustively reviewed.

## 2. Nevirapine

**Scheme 1:**
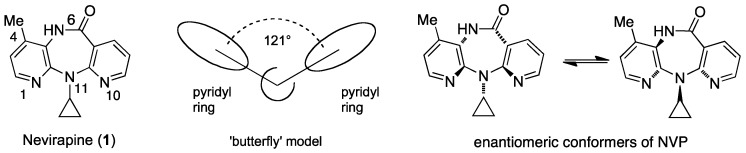
Butterfly model and enantiomeric conformers of nevirapine.

Nerivapine (**1**, NVP) (Viramune) was designed and synthesized by Merluzzi and co-workers at Boehringer Ingelheim Pharmaceutical Inc. [42,43] and approved in 1996 by the FDA for the treatment of AIDS/HIV infection in combination with NRTIs. NVP is well tolerated, but, like other NNRTIs, leads to rapid development of drug resistance due to drug-specific amino acid mutations in the NNBS of the RT [44,45,46]. Moreover, NVP causes cross resistance to drugs belonging to the NNRTI class. X-ray studies [47] of NVP revealed a butterfly-like conformation formed by intersection at an angle of 121° of two planes of up- or downwards pyridyl rings from the central diazepinone nucleus (Scheme 1). The pendant 11-cyclopropyl ring lays almost perpendicularly to the plane of the diazepinone, opposite to the two pyridyl rings [48]. The crystal structure shows small deformations of NVP, whereas the butterfly-like active conformation is preserved in the complexes of NVP with the HIV-1 RT [49]. The lack of plane of symmetry and the flexibility of the diazepinone allows NVP to exist as a mixture of atropisomeric enantiomers with relatively slow interconversion, despite the absence of stereogenic centers, in the presence of a quaternary stereogenic center by memory of chirality [50]. NVP displayed ^1^H-NMR spectrum signals characteristic of a chiral compound at room temperature. NMR and computational studies revealed that at room temperature the two enantiomeric conformations interconvert with a barrier of about 76 kJ·mol^−1^. Enantiomerisation half-life of NVP at room temperature is in the order of seconds, is not an atropisomeric compound, and therefore cannot exist as separable enantiomers [48].

Recently NVP was isolated from 100 kg seeds of *Cleome viscosa* (Capparidaceae) with a yield of 0.00397% [51]. It was suggested that plant might have biosynthesized NVP in response to either some abiotic stress or that the compound has been triggered in response to some kind of endogenous plant–pathogenic interaction. The crystal structure of the natural molecule was studied in order to confirm its structure unequivocally. UV, IR, NMR of natural and synthetic NVP were found to be identical. In contrast, the natural NVP isolated from the seeds of *Cleome viscosa* was shown to be different from synthetic NVP through isotope ratio mass spectrometric (IRMS) analysis. The chiral HPLC analysis of the natural and synthetic NVP showed a single peak in the HPLC chromatogram due to the rapid interconversion of the molecule at room temperature. The synthetic acetyl derivative of NVP at the carboxamide nitrogen showed two peaks in chiral HPLC confirming the racemic nature of NVP. The N5-acetyl-NVP showed atropisomeric behavior by increasing the barrier to the N inversion leading to the possibility of separating the two enantiomers.

## 3. Efavirenz

Delavirdine (Rescriptor) and efavirenz (**2**, EFV) (Sustiva) were successfully launched as anti-HIV-1 drugs in 1997 and 1998, respectively [52] (it should be noted that delavirdine is no longer used as a component of ART treatments). EFV is the first-line drug of initial combination regimes for the antiretroviral naive patients and as prophylaxis after exposure to HIV infection. EFV is potent and tolerated but its use is limited by its low genetic barrier to development of resistance [53]. Recently, EFV-based ART treatments have been classified in the alternative regimens category due to the development of serious CNS-related toxicities and a possible association with depression, aggressive behavior and suicidality [28].

**Scheme 2:**
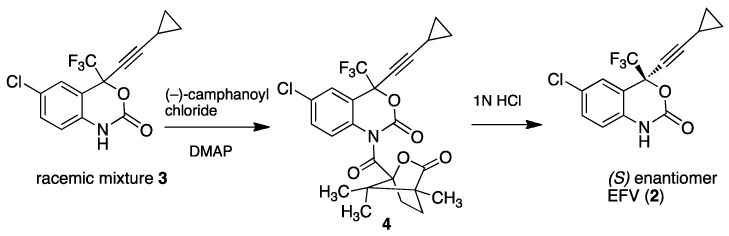
Early separation of (*S*)-efavirenz (EFV) from the racemic mixture **3**.

EFV (formerly L-743,726, DMP-266) was discovered by Merck Research Laboratories (West Point, PA, USA, and Rahway, NJ, USA) [54,55]. The absolute configuration of EFV was determined from a single crystal X-ray diffraction analysis of the (−)-camphanate imide derivative **4** of the racemate **3** (Scheme 2). Solving the structure resulted in the determination of the chiral site of the benzoxazin-2-one ring in the *S* configuration. The (*S*)-enantiomer was found to be a potent inhibitor of HIV-1 WT in MT-4 cell culture with IC_95_ in the 1.5–3.0 nM range. It also exhibited antiviral activity with 95% inhibitory concentrations of ≤1.5 µM against a panel of HIV-1 NNRTI-resistant mutant viruses carrying single amino acid substitution of the RT. Whereas, the (*R*)-enantiomer, obtained in a similar way, was inactive in the *in vitro* RT inhibition assay [54].

**Scheme 3:**
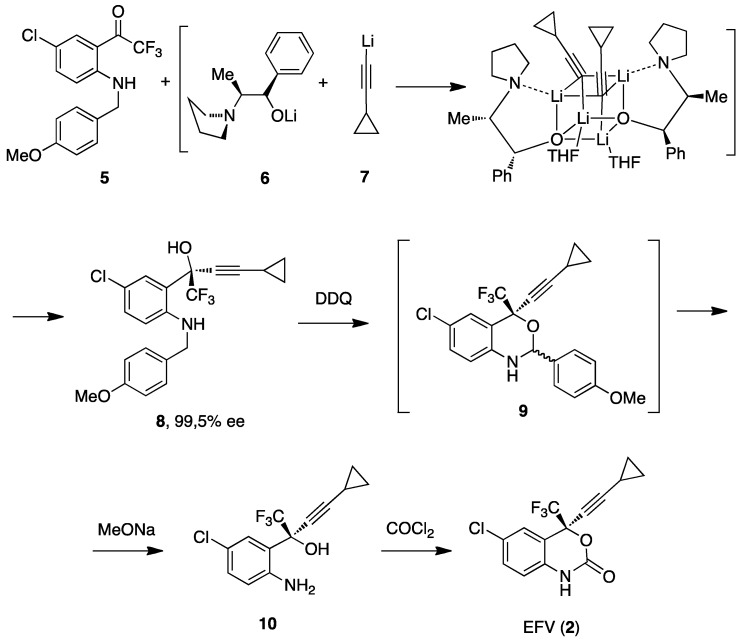
Thompson enatioselective synthesis of efavirenz.

Efficient synthetic processes for the production of EFV have been developed [56]. The presence of a stereo center in EFV prompted the synthesis of stereospecific synthesis. The asymmetric addition of acetylide to the trifluoromethylketone **5** was optimized by Thompson [57] by adding *n*-buthyl lithium to a solution of (1*R*,2*S*)-*N*-pyrrolidinylnorephinephrine [58] and cyclopropylacetylene. This reaction allowed the chiral complex to form that is required to obtain high (50:1) enantioselectivity. Two equivalents of lithium alkoxide **6** and two equivalents of lithium acetylide **7** were required to achieve full conversion. Mixtures of **6** and **7** reacted with **5** at low temperature to provide high enantioselectivities. Lithium cyclopropylacetylide and lithium alkoxide formed mixtures of tetramers [57]. The 4-methoxybenzyl group was removed with dichlorodicyanoquinone (DDQ) to afford quantitatively the amine **9** (11.5:1 distereomeric) which was transformed to amino alcohol **10** with sodium methoxide. Finally the benzoxazinone ring was easily obtained by reaction of **10** with phosgene to give EFV in excellent optical purity (enantiomeric excess (ee) >99.5%). This processed analytically pure EFV in an overall yield of 62%, (Scheme 3) [59].

**Scheme 4:**
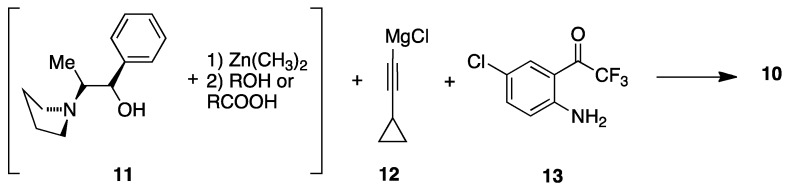
Chen enatioselective synthesis of **10**.

Alternatively, the chiral ligand **11** was treated with dimethylzinc followed by the addition of an alcohol or carboxylic acid [60]. This solution was treated with 1.2 eq of chloromagnesium acetylide **12** and then with **13**. The process provides EFV in kilogram scale in 95.2% yield and 99.3% ee (Scheme 4).

In 2013, Seetharamappa *et al.* reported the first chiral HPLC method for the separation of the (*R*)- and (*S*)-enantiomers of EFV [61] by using commercially available chiral stationary phase (Chiralcel OD-H). This efficient method was successfully applied for the evaluation of enantiomeric purity in bulk and pharmaceutical formulations, and it was sensitive at the low detection limit of 65 ng/mL for (*R*)-EFV. At the same time Singh *et al.* developed a rapid isocratic chiral ultra-performance liquid chromatography (UPLC) method for the separation of the EFV enantiomers [62]. Good resolution was achieved by normal phase UPLC using chiral column Chiracel OD-H (250 mm × 4.6 mm) 5 μm using *n*-hexane and isopropyl alcohol 9:1 as a mobile phase at column temperature of 30 °C and flow rate of 1.0 mL/min. This method was used to determine the amount of (*R*)-EFV in pure and pharmaceutical formulations of (*S*)-EFV and led to detect and quantitate (*R*)-EFV to the levels of 249 ng/mL and 75 ng/mL, respectively.

## 4. DuPont Compounds (DPC)

**Scheme 5:**
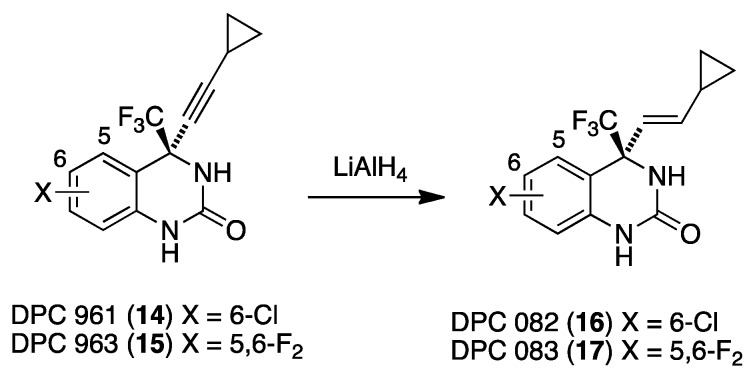
Quinazolinone analogues of EFV.

DuPont Pharm. Co. initiated a drug discovery program to identify expanded-spectrum NNRTIs which possess increased potency toward K103N mutation [63,64]. The K103N mutation is the major mutation detected in patients receiving EFV-containing treatments whose viral loads rebounded after an initial response to the drug [65,66]. Identification of the alkynylquinazolinones DPC 961 (**14**) and DPC 963 (**15**) and the alkenylquinazolinones DPC 082 (**16**) and DPC 083 (**17**) (Scheme 5) resulted in potent NNRTIs with low nanomolar potency against HIV-1 WT and the K103N and L100I single-mutation strains.

**Scheme 6:**
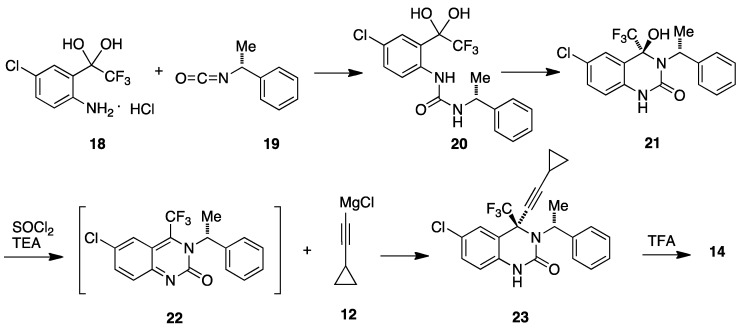
Magnus asymmetric synthesis of DPC 961.

The enantiomers **14** and **15** were separated from the corresponding racemates by chiral HPLC. The stereochemistry of **14** was determined using X-ray crystallography diffraction, and the absolute stereochemistry of DPC **15** was inferred from the antiviral and enzyme data. The asymmetric synthesis of **14** was achieved in three steps starting from keto-aniline **18** with an overall yield >55%. The asymmetry was induced by the chiral auxiliary (*R*)-(+)-α-methylbenzylamine, utilizing a new asymmetric 1,4-addition protocol [67]. The hemiaminal **21** in the presence of triethylamine (TEA) and thionyl chloride at 0 °C rapidly generated intermediate **22** which was trapped at −50 °C by an excess of **12**. The diastereomer **23** was obtained in 85% isolated yield and 92% diasteromeric excess. Finally, dihydroquinazolinone **23** was converted into **14** with trifluoroacetic acid (Scheme 6).

As compared to benzoxaxinones, in the quinazolinone series, the stereochemistry of the quaternary center is inverted [55,68]. Against the HIV-1 K103N mutant strain in HIV-1 infected cell assay, DPC derivatives **14** (K103N IC_90_ = 10 nM) and **15** (K103N IC_90_ = 11 nM) were superior to EFV (K103N IC_90_ = 64 nM), while the corresponding enantiomers exhibited virtually no activity. The olefin derivatives **16** and **17** were as potent as **14** and **15**, and all were either as potent as, or more potent than, EFV. Most importantly, this potency profile was maintained against a panel of single- and double-HIV-1 strains carrying the K103N mutation [63].

A chiral method was developed to resolve the racemic mixtures and control the enantiomeric purity of compounds **14** and **17** and to monitor for any chiral inversion in the drug substance and in the tablet formulation during stability studies [69]. Chiralpak AD, Chirobiotic V, and Whelk-O columns were evaluated with various nonaqueous mobile phases which were selected because of poor water solubility of **14** and **17**. The order of elution on the Whelk-O and the Chiralpak AD columns was found to be affected by the degree of unsaturation of α carbon–carbon bond on the side chain, while the stereoselectivity was negatively affected by the presence of an NH group next to the chiral center. This method was able to detect the enantiomeric purity of **14** and monitor chiral inversion on stability. Chirobiotic V column showed high stereoselectivity for **17** and specificity toward nonenantiomeric impurities using a mobile phase composed of acetonitrile and methanol 90:10 (*v*/*v*).

## 5. DAPY

**Scheme 7:**
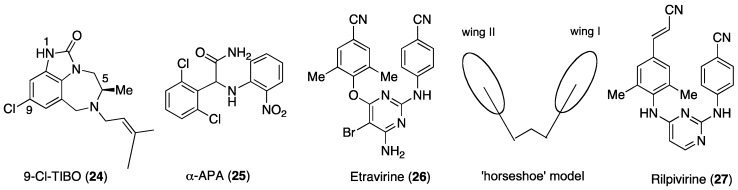
Structure of tetrahydro-imidazo-benzodiazepin-one (TIBO), α-anilino-phenyl-acetamide (α-APA), Etravirine (ETR) and Rilpivirine (RPV), and horseshoe model.

The story of the DAPY family started in 1987 with tetrahydro-imidazo-benzodiazepin-one (TIBO) derivatives [20]. Despite notable chemical differences from other NNRTIs, the bound conformation Cl-TIBO into the NNBS of the RT shared similarities to other NNRTIs [70]. For example, the 5-methyl group of 9-Cl-TIBO (**24**) (Scheme 7) achieved spatial equivalence to the cyclopropyl group in NVP (see Scheme 1) and the amide in α-APA and methyl in HEPT. Such spatial equivalence was achieved by the (+)-(*S*)-Me enantiomer of 9-Cl-TIBO which was in agreement with the inspected stereospecificity specification [20].

After development, TIBO was discontinued due to complex chemical synthesis and poor bioavailability, a new class of α-anilinophenylacetamido (α-APA) derivatives was discovered in 1991 by random screening [71]. A program of synthesis was subsequently initiated, aimed at enhancing the anti-HIV potency of α-APA derivatives. The antiretroviral activity of α-APA derivatives was clearly stereospecific. For example, (−)-(*R*)-**25** (EC_50_ HIV-1_IIIB_ = 33 nM in MT-4 infected cells) was 50-fold more potent than the corresponding (+)-(*S*)-isomer (EC_50_ = 1700 nM). The stereospecificity seen for the enantiomers of (−)-(*R*)-**25** extended to derivatives of this series [72].

In 1993 evolution of α-APA to improve the spectrum against the HIV-1 mutant strains led to the discovery of the extremely active imidoyl thiourea (ITU) class [73]. Incorporation of a cyanoguanidine moiety resulted in diaryltrazine (DATA) derivatives which showed subnanomolar potency *vs.* the wild-type HIV-1 (LAI strain) and low nanomolar potency against a battery of clinically important HIV-1 mutants, but were inactive against the double mutant strains K103N-L100I and K103N-Y181C [73]. As a further advance, the central triazine core was replaced with a pyrimidine ring and the 2,6-dichlorobenzyl moiety with 2,4,6-trisubstituted aromatic groups to give the DAPY series [74]. Chemical manipulation on the DAPY scaffold resulted in the discovery of etravirine (ETR) (**26**) and rilpivirine (RPV) (**27**) (Scheme 7), two broad spectrum NNRTIs with good metabolic stability and plasma levels. ETR (Intelence) and RPV (Edurant) were launched onto the market in 2008 and 2011, respectively. Crystallographic studies showed that ETR in complex with the HIV-1 RT adopted the “horseshoe” binding conformation [75]. The dynamic adaptation of DAPY analogues into the NNBS of the HIV-1 RT might explain for their ability to inhibit HIV-1 RT carrying resistance mutations [76].

**Scheme 8:**
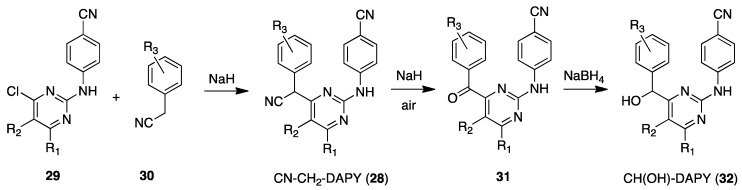
CN-CH_2_-DAPY and CH(OH)-DAPY derivatives.

Chemical studies to improve the antiretroviral properties of DAPY compounds focused on wing II, since wing I proved to be an indispensable pharmacophore. The influence of a cyano substituent on the CH_2_ linkage to give CN-DAPYs was investigated [77]. The CN-CH_2_-DAPYs **28** which were synthesized by condensation of **29** [78] with an appropriately substituted arylacetonitrile **30** in the presence of sodium hydride, were assayed as racemic mixture (Scheme 8). Most derivatives inhibited the HIV-1 WT replication in the nanomolar range of concentration ((±)-**28a**: R_1_ = H, R_2_ = Me, R_3_ = 2-F, EC_50_ HIV-1_IIIB_ = 1.8 nM in MT-4 infected cells; EFV as reference compound: EC_50_ HIV-1_IIIB_ = 3.5 nM) [77]. Further introduction of a hydrophilic hydroxyl group to the CH_2_ linker led to a structurally novel CH(OH)-DAPYs **32**. The synthesis of derivatives **32** was performed by air oxidation of **28** in presence of NaH and subsequent NaBH_4_ reduction of the corresponding ketones **31** [79]. The most active CH(OH)-DAPY compound ((±)-**32a**: R_1_ = R_2_ = H, R_3_ = 2-Cl) inhibited the HIV-1 WT with an EC_50_ value 9 nM in MT-4 cells and the double K103N-Y181C HIV-1 mutant strain with EC_50_ = of 6200 nM (EFV: EC_50_ HIV-1_IIIB_ = 5 nM; EC_50_ HIV-1 K103N-Y181C = 443 nM) [80]. The racemate **32a** was successfully separated into the corresponding (+) and (−) enantiomers by supercritical fluid chromatography (SCF) with ee >99% and purity >99%. Their absolute (*R*) and (*S*) configuration, respectively, was assigned by the experimental electronic circular dichroism (ECD) spectrum and simulated ECD spectra calculated by time-dependent density functional theory (TDDFT) calculations. The (+)-(*R*)-**32a** enantiomer inhibited the HIV-1 WT with an EC_50_ of 5 nM and was 13-fold superior to the (−)-(*S*)-**32a** enantiomer. In contrast, the (−)-(*S*)-**32a** enantiomer was more potent than (+)-(*R*)-**32a** against the HIV-1 K103N-Y181 mutant strain and HIV-1 ROD strain (EFV: EC_50_ HIV-1_IIIB_ = 5 nM, K103N-Y181C = 440 nM) [80]. According to the biological results, docking studies highlighted different binding poses of the two enantiomers into the NNBS of the HIV-1 RT.

## 6. DABO and DAPY-DABO Hybrids

**Scheme 9:**
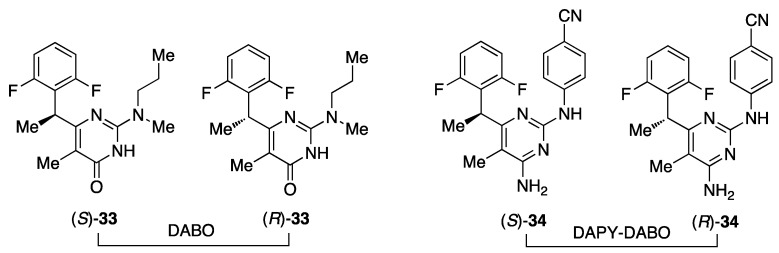
DABO and DABO-DAPY hybrid derivatives.

The dihydroalkyloxybenzyloxopyrimidine (DABO) family of HIV-1 NNRTIs have been developed since 1992 [81]. As a further development, a series of DAPY-DABO hybrid compounds was synthesized by linking the 4-cyanoaniline group typical in DAPY at the position 2 of the pyrimidine core and the 2,6-difluorobenzyl group peculiar in DABO at the position 6. These compounds were characterized by the presence at position 4 of the pyrimidine of characteristic groups either DAPY or DABO series. The DAPY-DABO hybrids showed nanomolar anti-HIV-1 activity in both enzymatic and cellular assays [82]. Two racemic mixtures among the DABO and DAPY-DABO hybrids families were separated by chiral HPLC on coated-type Chiralpak AD chiral stationary phase (CSP) using ethanol containing 0.1% of diethylamine as eluent. Because of unsuccessful X-ray crystal efforts, the stereochemical information on the four chiral compounds was achieved by analyzing their chiroptical properties. The (*S*) configuration was assigned to the first-eluted dextrorotatory enantiomers, (+)-(*S*)-**33** and (+)-(*S*)-**33**, and the (*R*) configuration was assigned to the second-eluted levorotatory (−)-(*R*)-**34** and (−)-(*R*)-**34** counterparts [82] (Scheme 9). In both cellular and enzyme assays, the (*R*)-**33** and (*R*)-**34** enantiomers were significantly superior to the (*S*)-ounterparts (the racemates showed intermediate potencies). Compound (*R*)-**34** (EC_50_ HIV-1_NL4-3_ = 0.1 nM; EC_50_ K103N = 0.8 nM; EC_50_ Y181C = 31 nM; EC_50_ Y188L = 7.1 nM; MT-4 infected cells; EFV: EC_50_ HIV-1_NL4-3_ = 7.3 nM, EC_50_ K103N = 340 nM; EC_50_ Y181C = 10 nM; EC_50_ Y188L = 1600 nM) [83] exhibited better profile than (*R*)-**33** in inhibiting the cytopathic effect of HIV-1 strains in MT-4 cells. However, (*R*)-**33** was generally less toxics reaching higher selectivity index than (*R*)-**34**. In the RT enzymatic assays, (*R*)-**34** was superior to (*R*)-**33** against the HIV-1 WT, K103N, V106A, and Y188L RTs, while (*R*)-**33** more potent against the L100I and Y181I RTs.

## 7. PETT

**Scheme 10:**
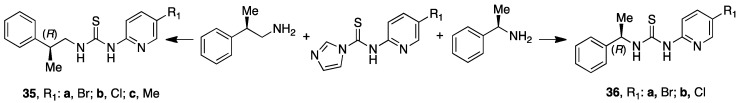
β-methyl-PETT and α-methylbenzylthiourea derivatives.

Phenethylthiazolylthiourea (PETT) HIV-1 NNRTI class was discovered by Bell [84] and Cantrell [85] at Lilly research laboratories. In 2004, Uckun reported a number of chiral thiourea derivatives in order to examine the effect of stereochemistry of these PETT derivatives on HIV-activity [86,87]. A series of chiral PETT derivatives was prepared by condensing thiocarbaimidazole derivatives with the respective chiral amines in anhydrous DMF. β-methyl phenethylthiazolylthiourea derivatives (*R*)-**35a**–**c** with halogen (Br, Cl) or methyl substitutions respectively at 5-position of the pyridine were the most potent against the HIV-1 rRT *in vitro* with nanomolar inhibitory concentrations. (Scheme 10). On the contrary, the (*S*)-anantiomers showed lower inhibition in the micromolar range of concentration. Similarly, only β-methyl thiazolyl derivatives of the (*R*) series exhibited anti-HIV activity. (*R*)-**35a**–**c** compounds inhibited the HIV-1 strain HTLV_IIIB_ in human PBMCs with EC_50_ values of 3 nM, <1 nM and 3 nM, respectively (EFV: EC_50_ HIV-1_IIIB_ = 1.5 nM) [86]. Molecular modeling studies showed that the (*R*) stereoisomers of chiral halopyridyl and thiazolylthiourea compounds would fit the NNBS of the HIV-1-RT better than their (*S*) enantiomers, mainly due to unfavorable interactions of the (*S*) compounds in the pocket of the NNBS surrounding the Y181. In agreement with the β-methyl series, all the (*R*) enantiomers of the chiral α-methylbenzylthiourea derivatives proved to be potent inhibitors of the HIV-1, whereas their (*S*) counterparts were inactive. Against the HIV-1 rRT compound **36a** achieved an activity of 1.6 µM and **36b** of 1.2 µM. Both compounds inhibited the HIV-1 HTLV_IIIB_ with EC_50_ of 10 nM; NVP: EC_50_ = 34 nM [87].

## 8. IAS

The indolylarylsulfone (IAS) story started in 1993, when Merck reported L-737,126 as a potent and selective HIV-1 NNRTI with EC_50_ = 1 nM against HIV-1_IIIB_ WT [88]. SAR studies of the IAS class showed that the methyl groups at the positions 3′,5′ of the 3-phenylsulfonyl moiety of the indole are a key structural requirement for an effective inhibition of the HIV-1 mutant strains [89]. IAS derivatives containing a chiral center were synthesized by coupling of glycine/alanine amino acid units to the 2-carboxyamide function. The aim was to improve the chemical interactions inside the NNBS of the HIV-1 RT by remodeling of the substituents at indole-2-sidechain [90]. The initial results prompted the synthesis of a new series of peptide-IAS derivatives by coupling of natural and unnatural amino acids to the indole-2-carboxamide [91]. Such IASs exhibited potent inhibition of HIV-1 with inhibitory activities comparable to EFV in CEM cells. To evaluate the influence of the chiral center at the amino acid unit, three D,L racemic mixtures were separated by chiral HPLC. Against HIV-1 WT RT, the L and D enantiomers from the corresponding racemates showed negligible differences of activity. Against the K103N mutation, two d-enantiomers were about five times more potent than the corresponding l-enantiomers, while the third couple of enantiomers did not show any significant difference of activity [91].

**Scheme 11:**
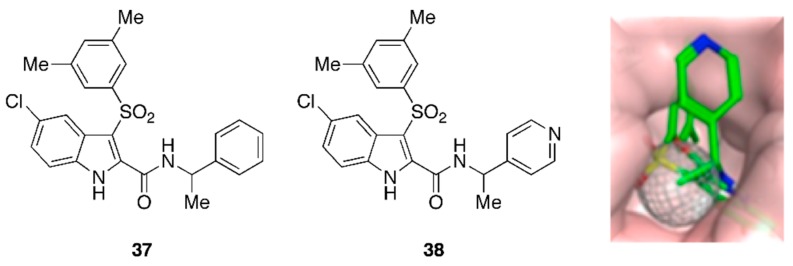
Structures of IASs **37** and **38**. Binding mode of (*R*)-**38** (green) into the NNBS of the K103N RT: the surface shows shape and position of the methyl group following the induced fit model.

A new series of IAS derivatives was synthesized by coupling of the 2-carboxyamide with appropriate benzyl- or phenylethylamines. These new IASs shared similar features that are specific of ETR the presence of a pendant (third) aromatic ring [76]. To evaluate the influence of the asymmetric center at the 1-phenylethyl moiety, the racemic mixture (*R*,*S*)-**37** was separated using a chiral HPLC equipped with the stainless-steel Chiralcel OD (250 mm × 4.6 mm i.d. and 250 mm × 10 mm i.d.) to give the pure enantiomers (*R*)-**37** and (*S*)-**37** (Scheme 11) [92]. Against the HIV-1_NL4-3_ strain, the enantiomers (*R*) and (*S*) showed small differences of activity. In contrast, (*R*)-**37** (EC_50_ K103N = 4.3 nM; EC_50_ Y181C = 86 nM; EC_50_ Y188L = 193 nM; EFV: EC_50_ K103N = 130 nM; EC_50_ Y181C = 160 nM; EC_50_ Y188L = 760 nM, MT-4 infected cells) [93] was significantly more potent than (*S*)-**37** against the panel of mutant HIV-1 strains: 30-fold *vs.* the K103N, 40-fold *vs.* Y181C, >189-fold *vs.* Y188L, and >22-fold *vs.* K103N-Y181C. Against the HIV-1 RTs, the enantiomers (*R*)-**37** and (*S*)-**37** were almost equipotent against the HIV-1 WT RT, and (*R*)-**37** (IC_50_ = 90 nM) was 104-fold more potent than (*S*)-**37** against the HIV-1 K103N mutated RT. Docking studies suggested that the difference in the observed inhibitory activities of (*R*)-**37** and (*S*)-**37** could be due to kinetic rather than affinity differences: while (*R*)-**37** was able to seal the binding pocket, (*S*)-**37** left the site accessible to water, leading to a negative effect on the binding kinetic of this inhibitor.

A new series of IAS derivatives was designed addressing the strategy of the additional third cyclic moiety [93]. The racemic mixture (*R*,*S*)-**38** was directly separated by enantioselective HPLC using the cellulose derived Chiralcel OD chiral stationary phase (CSP) and *n*-hexane/ethanol 1:1 as a mobile phase. The optimized analytical enantioselective method was scaled-up to a semipreparative level to obtain milligram amounts of the pure enantiomers for screening. The stereochemical characterization of (*S*)-**38** and (*R*)-**38** was performed by circular dichroism (CD) correlation using the (*R*)-**37** and (*S*)-**37** pure enantiomers as reference samples. The (*R*) configuration was empirically assigned to the more retained enantiomer on the Chiralcel OD CSP, and the (*S*) configuration, to the less retained enantiomer. The enantiomers (*S*)-**38** and (*R*)-**38** were equipotent (EC_50_ = 0.2 nM, MT-4 infected cells) against the HIV-1_NL4-3_ WT strain. Whereas, (*R*)-**38** was more potent than (*S*)-**38** against the HIV-1 mutant strains K103N (EC_50_ = 0.2 nM), Y181C (EC_50_ = 2.1 nM) and K103N-Y181C (EC_50_ = 150 nM; EFV: EC_50_ K103N = 130 nM; EC_50_ Y181C = 160 nM, EC_50_ K103N-Y181C >317) [94]. Compound (*R*)-**38** was remarkably superior to NVP and EFV, and superior to AZT against the WT, K103N, and Y181C strains. Docking experiments showed some significant differences in the binding modes (*R*)-**38** and (*S*)-**38** in the NNBS of the HIV-1 K103N mutated RT. The methyl group of (*R*)-**38** pointed toward the cleft created by the K103N mutation, sealing the binding pocket and reducing the solvent-accessible surface, while the corresponding group of the (*S*)-**38** enantiomer left the pocket more exposed to solvent. Molecular dynamics simulation showed stable trajectories for both enantiomers. However, calculation of the solvent-accessible surface area (SASA) [94] showed significant differences for the K103N RT/(*S*)-**38** and K103N RT/(*R*)-**38** complexes. In particular, SASA of the receptor in complex with the (*S*) enantiomer was greater than the corresponding one for the (*R*) enantiomer, reporting values of 235.64 and 210.20 Å^2^, respectively. Inside the NNBS the (*S*) enantiomer showed a number of water molecules surrounding the methyl cleft, 3.5 times greater than the number of solvent molecules observed for the (*R*) enantiomer. These results provided a justification for the different biological activity observed between (*R*)-**38** and (*S*)-**38** (Scheme 11) [93].

## 9. API

**Scheme 12:**
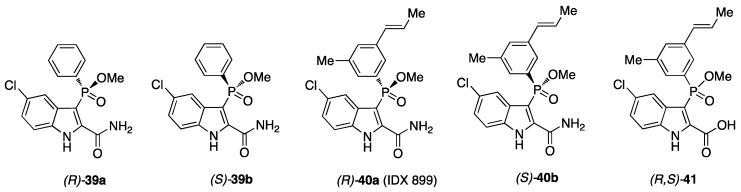
Arylphosphoindole (API) derivatives.

A new series of arylphosphoindole (API) derivatives was designed by Idenix Laboratories by replacing the 3-sulfonyl bridging group of IAS NNRTIs with a phosphinic acid methyl ester group [95]. Compound API **39** as a racemic mixture showed low nanomolar activity against the HIV-1 WT, K103N, Y181C strains and reasonable activity against the HIV-1 K103N-Y181C double mutant strains (Scheme 12). Chiral separation of the racemate **39** by supercritical fluid chromatography (SFC) afforded the enantiomers (*R*)-**39a** and (*S*)-**39b**. The analytical separation was performed on Chiralcel OD-H (250 mm × 4.6 mm) column using *n*-heptane/ethanol/diethylamine 70/30/0.1 as the mobile phase, flow rate 1.0 mL/min. At preparative level was used a Chiralpak AD (250 mm × 20 mm) column with CO_2_/MeOH + 1% diethylamine 80/20 as the mobile phase, flow rate 60 mL/min., flow rate 62 mL/min. The enantiomer **39a** was superior to the racemate **39** against the HIV-1 WT and the mutant viruses (HIV-1 WT, EC_50_ = 0.1 nM; K103N, EC_50_ = 1.2 nM, Y181C, EC_50_ = 3.6 nM; K103N-Y181C, EC_50_ = 137.4 nM, MT-4 infected cells). In contrast, **39b** was remarkably less potent against HIV-1 WT and did not inhibit the HIV-1 NNRTI drug-resistant strains. The stereochemistry **39a** was assigned to be (*R*) according to molecular modeling studies (binding energy: **39a** = −23 kcal/mol; **39b**: −18 kcal/mol). The (*R*) stereochemistry of **39a** was confirmed using X-ray crystallography diffraction.

In order to achieve broad spectrum against the HIV-1 drug resistant mutant strains, Idenix introduced the Z-cyanovinyl typical in RPV at position 3′ of the 3-phenyl ring of API derivatives. Among these compounds, the enantiomer (*R*)-**40a** (IDX 899) (Scheme 12) potently and selectively inhibited HIV-1 WT and NNRTI drug resistant HIV-1 mutant strains *in vitro*, and was selected for clinical studies. In 2009, Idenix granted GlaxoSmithKline exclusive worldwide rights to IDX 899. Preclinical data for IDX-899 suggested a significantly greater barrier to resistance compared with EFV. IDX 899 effectively reduced HIV-1 RNA levels and increased CD4+ cell counts in treatment-naïve patients infected with HIV-1 during phase I clinical trials [96].

Separation of the racemic (*R*,*S*)-carboxylic acids **41** was performed on hundred grams scale by a two steps procedure to obtain the required enantiomer [96]. Treatment of **41** with (−)-cinchonidine in acetone furnished the corresponding diastereomeric mixture. The precipitated solid was isolated by filtration. The filtrate was concentrated, and both solid and residue from the filtrate were cleaved with 1 N HCl and ethyl acetate. The overall recovery of enantiomeric pure acid was 70%. An alternate separation of the racemate **41** was performed using (1*R*,2*S*)-ephedrine and (1*S*,2*R*)-ephedrine monohydrate in ethanol. The basis of this separation was the same as that described with (−)-cinchonidine.

**Scheme 13:**
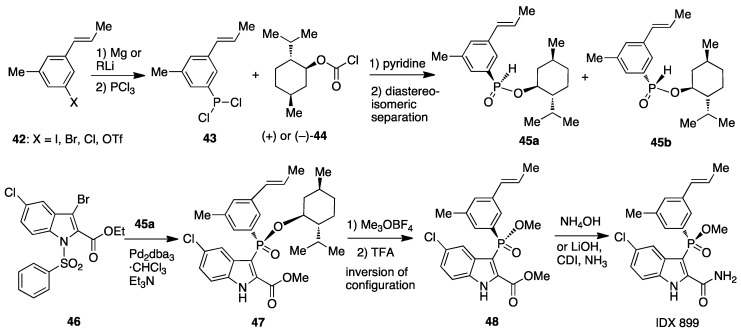
Enantiosynthesis of IDX 899.

The enantiosynthesis [97] of IDX 899 required the preparation of menthyl phosphinate compound from aryl halides or triflates **42** in the presence of Mg or RLi and PCl_3_ to give dichlorophosphite derivative **43**. Treatment of **43** with (+)- or (−)-menthylchloroformate **44** in pyridine according to the Hewitt [98] reaction afforded a mixture of diastereoisomers which was separated into **45a** and **45b** by crystallization at low temperature in hexane. Reaction of **45a** with ethyl 3-bromo-5-chloro-1-phenylsulfonyl-1*H*-indole-2-carboxylate (**46**) in the presence of tris(dibenzylideneacetone) dipalladium(0)-chloroform adduct (Pd_2_dba_3_·CHCl_3_) and triethylamine afforded **47**. Reaction of **47** with trimethyloxonium tetrafluoroborate (Meerwein salt) and trifluorocaetic acid with inversion of configuration afforded **48**. Finally, ester **48** was transformed into IDX 899 by reaction with ammonium hydroxide in methanol or hydrolysis of the ester to carboxylic acid with lithium hydroxide, activation with carbonyl diimidazole as subsequent displacement of the imidazolide with ammonia (Scheme 13).

## 10. TFMI

**Scheme 14:**
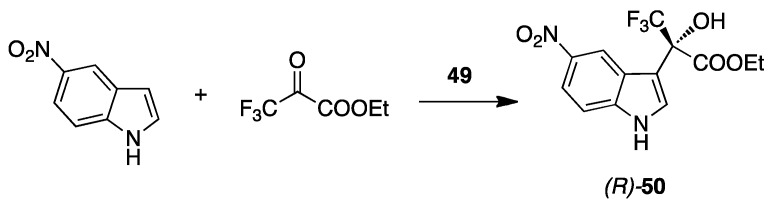
CSCS catalyzed enantiosynthesis of (*R*)-**50**.

A series of non-racemic trifluoromethylated indoles (TFMIs) were synthesized as HIV-1 NNRTIs and assayed by a TZM-bl cell assay [99]. The TFMIs were synthesized using an asymmetric Friedel–Crafts reaction of indoles with alkyl trifluoropyruvates catalyzed by C3-symmetric cinchonine-squaramide (CSCS, **49**) organocatalyst that was developed by the same authors [100] (Scheme 14). The chiral trifluoromethylated indoles were obtained in excellent yield and enantioselectivity up to >99% ee. With few exceptions, the chirality of the molecules played a key role on the antiretroviral activity. As HIV-1 inhibitors the (*R*)-enantiomers were superior to the corresponding (*S*)-enantiomers, and the increased activity of the (*R*)- over the (*S*)-enantiomer ranged from 6 to 32-fold. Compound (*R*)-**50**, with a 5-NO_2_ substituent, was the most potent HIV-1_IIIB_ inhibitor in the TZM-bl cells, with an EC_50_ value of 19 nM (EFV EC_50_ = 15 nM), CC_50_ value of 210.697 μM and SI (selectivity index, CC_50_/EC_50_) value of 11 089, respectively [99].

## 11. Conclusions

Chirality is recognized to considerably affect the pharmacological profile of drugs due to the highly specific interaction of the ligand with the recognition site. Accordingly, the enantioselectivity plays an important role for the binding of antiviral agents to HIV-1 RT. Among chiral HIV-1 NNRTIs, one enantiomer is often superior to its corresponding counterpart against the HIV-1 WT and the HIV-1 drug resistant mutant strains. This review illustrates the various studies made to investigate the effect of chirality on the activity of HIV-1 NNRTIs. Chiral HIV-1 NNRTIs were achieved by either chiral separation or enantiosynthesis. This review may assist in a more in depth understanding of the mechanism of action of HIV-1 NNRTIs, as well as suggest concepts for the design and synthesis of new generation HIV-1 NNRTI agents.
